# Management and intensity of medical end‐of‐life care in people with colorectal cancer during the year before their death in 2015: A French national observational study

**DOI:** 10.1002/cam4.2527

**Published:** 2019-09-25

**Authors:** Audrey Tanguy‐Melac, Anne‐Sophie Aguade, Anne Fagot‐Campagna, Christelle Gastaldi‐Ménager, Jean‐Marc Sabaté, Philippe Tuppin

**Affiliations:** ^1^ Caisse Nationale d'Assurance Maladie (Cnam) – Direction de la stratégie des études et des statistiques Paris France; ^2^ Service de Gastroentérologie Hôpital Avicenne AP‐HP Bobigny France; ^3^ INSERM U‐987 Physiopathologie et Pharmacologie Clinique de la Douleur Hôpital Ambroise Paré Boulogne‐Billancourt France

**Keywords:** cause of death, colorectal cancer, end of life, France, healthcare use, medical cost, palliative care

## Abstract

The care pathway of patients with colorectal cancer (CRC) 1 year prior to death, their causes of death and the healthcare use, and associated expenditure remain poorly described together. People managed for CRC (2014‐2015), covered by the national health insurance general scheme and who died in 2015 were selected from the national health data system. A total of 15 361 individuals (mean age: 75 years, SD: 12.5 years) were included, almost 66% of whom died in short‐stay hospital (SSH), 9% in hospital at home (HaH), 4% in rehabilitation units (Rehab), 6% in skilled nursing homes (SNH), and 15% at home. At least one other cancer was identified for one‐third of these people. Almost one‐half of people presented cardiovascular comorbidity, 21% had chronic respiratory disease, and 13% had a neurological or degenerative disease. During the last month of life, 83% were admitted at least once to SSH, 39% had at least one emergency department admission, 17% were admitted to an intensive care unit, 15% received at least one chemotherapy session (<60 years: 27%), and 5% received oral chemotherapy. Eighty‐eight percent of the 60% of individuals who received hospital palliative care (HPC) vs 75% of those without HPC were admitted to SSH at least once during the last month. Cancer was the main cause of death for 84% (SSH: 85%, home: 77%) and corresponded to CRC for 64% of them. The mean annual expenditure per person during the last year of life was €43 398 (SSH: €48 804). This study suggests a relatively high level of HPC use during the year before death for people with CRC in France. High rates of emergency department, intensive care, and chemotherapy use were observed during the last month of life. However, management is very largely SSH‐based with a small proportion of deaths at home.

## Graphical abstract

One of the objectives of this study was to describe hospital care pathways in people managed for CRC in France before their death in 2015. These flows were greater during the last 2 months of life, predominantly corresponding to transfers from home to acute wards and, to a lesser degree, from acute wards to Rehab. By focusing on the last 28 days of life, flows from home to acute wards increased before death, especially during the last week: 54% of people were still at home at the beginning of the last month of life and 15% were still at home at the time of death.

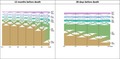

## 1 INTRODUCTION

The end‐of‐life pathway of people with certain cancers has been described as a gradual decline over several years, followed by a rapid and marked decline during the last weeks or months of life.^1^ Early palliative care (PC) provides patients with improved comfort and quality of life at the end of life.^2^, ^3^ Studies using quality indicators designed to measure the intensity of medical care at the end of life have reported inappropriate and “aggressive” health‐care use during the month before death, with frequent hospitalizations and emergency department or intensive care admissions and extensive use of chemotherapy.^4^, ^5^, ^6^ The development of PC in France and in other southern European countries has been described to lag behind that of English‐speaking countries.^7^ Nevertheless, a recent study, mainly on European countries, pointed a wide variation in palliative care services and patients across Europe and concluded that detailed characterization is the first step in improving PC services and research.^8^ In France, a new national plan (2015‐2018) for the development of end‐of‐life palliative care and supportive care was launched.^9^ This plan focussed on the following four areas: informing patients of their rights and ensuring person‐centered care and decision‐making; developing palliative care in the community; addressing regional disparities of access to palliative care services; and improving health‐care professionals' palliative care skills through education and clinical placements, and supporting research in palliative care. Nevertheless, few individual data were available and analyzed together. About colorectal cancer (CRC), there is a lack of data available for the end‐of‐life care pathways and intensity of medical care, their causes of death and associated expenditure, and they are mainly derived from studies based on small sample sizes.^10^, ^11^, ^12^, ^13^, ^14^, ^15^, ^16^, ^17^ The incidence of CRC in France was estimated to be 23 200 cases in 2018 with 9200 deaths (46% of women each).^18^ The net 1‐year, 3‐year, and 5‐year survivals for individuals diagnosed with CRC between 2005 and 2010 were 83%, 70%, and 64%, respectively.^19^ The annual cost for national health insurance of patients managed for CRC in France in 2014 was €1477 million, that is 11% of all cancers and 1.1% of total reimbursed expenditure.^20^ This cost was estimated by using the *Système National des Données de Santé* (SNDS) [National Health Data System], which is gradually being deployed in France. This database includes, for each individual, reimbursed hospital, and outpatient health‐care consumption data, including hospital palliative care (HPC), as well as causes of death.^21^, ^22^

The objective of this study, based on SNDS data, was to describe, in people managed for CRC in France, 1 year and 1 month before their death in 2015, their characteristics and comorbidities, hospital care pathways, and intensity of medical care, including HPC, their causes of death, and the expenditure reimbursed by national health insurance.

## 2 SUBJECTS AND METHODS

### 2.1 Setting

Of the 66 million inhabitants in France at the end of 2015, the general scheme covers salaried employees of the private sector and their dependents (ie, about 77% of the population living in France), as well as people covered by *Sections Locales Mutualistes* (SLM) [local mutualist sections], essentially civil servants, employees of territorial collectivities and public hospitals and students, that is about 11% of the population. Other schemes cover the rest of the population.^21^

### 2.2 Data source

The SNDS comprehensively collects individual outpatient data (age, sex, etc), as well as health‐care prescriptions and procedures reimbursed by French national health insurance, but it does not provide any clinical data concerning the results of physician visits, prescriptions or examinations. Nevertheless, it includes information on the presence of long‐term chronic diseases (LTD) (*Affection de Longue Durée*, ALD) eligible for 100% reimbursement of health‐care expenditure, when requested by the patient's general practitioner and after approval by the health insurance medical consultant. All this information is linked, via the national hospital discharge database (*Programme de médicalisation des systèmes d'information*: PMSI), to data concerning public and private hospital stays: short‐stay hospitals (SSH), Rehabilitation (Rehab), and hospital at home (HaH) and a specific database indicating whether or not the person is a resident of a skilled nursing home (SNH). Hospital discharge diagnoses and LTD diagnoses are coded according to the International Classification of Diseases 10th revision (ICD 10). Primary and secondary causes of death (ICD 10) are collected and analyzed by the Epidemiology Centre on Medical Causes of Death (Inserm‐CépiDc). These data are linked in the SNDS using an indirect matching procedure, prior to the introduction of a common identifier. The overall matching rate was 90% in 2015.

### 2.3 Identification of cases

The *Caisse Nationale d'Assurance Maladie* (CNAM), the general health scheme fund, has developed a tool based on SNDS data with algorithms designed to identify beneficiaries reimbursed for chronic diseases and common, serious or expensive diseases, and treatments each year, in order to study these diseases in terms of numbers, prevalence and incidence rates, expenditure and annual growth.^21^, ^22^, ^23^ Algorithms identify 56 nonexclusive groups of diseases, classified into 13 main categories, based on principal diagnoses, related or significantly associated diagnoses in short‐stay hospitals and psychiatric hospitals, LTD, dispensing of specific drugs, and specific procedures. In this tool, algorithms designed to identify people on cardiovascular prevention drugs (antihypertensives or lipid‐lowering drugs) or psychotropic drugs are considered on the basis of three annual reimbursements. Cancer is defined by short‐stay hospitalizations over a 5‐year period and/or LTD status based on specific cancer diagnoses. CRC cases are distinguished according to the presence of active treatment or surveillance. Cases with active treatment are defined as those requiring, over a 2‐year period, either hospitalization for treatment, with the exception of hospitalizations for assessment only, or hospitalization for metastasis, or initiation of management for an LTD, or treatment with certain specific therapies indicated in 2015 (ATC codes G03HA01, L01CD04, L01XX11, L02AA01, L02AA04, L02AE01, L02AE02, L02AE03, L02AE04, L02AE05, L02BB01, L02BB02, L02BB03, L02BB04, L02BX02, L02BX03, V10BX01, V10XX). Actively treated cancers were included in preference to cancers under surveillance.

The CNAM, as a health research institute, has permanent access to the SNDS database approved by decree and the French data protection authority (*Commission Nationale de l'Informatique et des Libertés*).

### 2.4 Study population

This retrospective observational study concerned all French national health insurance general scheme beneficiaries, who died in 2015 and who were identified by colorectal cancer treated in 2014 or 2015. The study was confined to these schemes because, at this time, the other schemes did not systematically record the vital status of their beneficiaries.

### 2.5 Description of variables

All data used for this study 1 year prior to death were derived from SNDS. In France, palliative care is provided by various types of hospitals or institutions: SSH have acute wards and palliative care facilities (specific wards, mobile teams providing palliative care advice and expertise to other health‐care professionals in other wards) and specific beds; rehab hospitals, to which people are usually admitted after an acute hospital stay and which are devoted to rehabilitation as well as palliative care depending on their rehabilitation specialization; and HaH care delivered at home by hospital teams. Data concerning HaH were analyzed separately, as HaH constitutes a specific type of management. Palliative care is also provided at home and in skilled nursing homes (SNH) by ambulatory teams not attached to hospital units, but this information is not available in the SNDS. In this study, the concept of HPC comprises palliative care delivered during the year, either before death or at the time of death, as these two types of HPC can be differentiated.

Chemotherapy not administered by the IV route consisted of drugs dispensed by a retail pharmacy and reimbursed by national health insurance, including subcutaneous injections using ATC codes (L01 to L04: antineoplastic, immunomodulating agents and endocrine therapy). Information on the place of death is available for deaths occurring during hospital stays (SSH, Rehab or HaH) or in SNH. However, among “other places” of death, death at home cannot be distinguished from death outside home: in a retirement home, in public places, etc.

### 2.6 Data and statistical analysis

For all health‐care benefits (drugs, clinical pathology procedures, consultations, etc) reimbursed by French national health insurance, SNDS indicates, in particular, the sums corresponding to the expenditure billed to the beneficiary, the reimbursable expenditure (ie, reimbursement basis) and the sum reimbursed to the beneficiary. However, drugs dispensed during a hospital stay are not individually reimbursed and were consequently not included in this study except for those billed by the hospital in addition to diagnosis‐related groups funding.

Data are expressed as the mean ± standard deviation (SD). The rates of at least one health‐care reimbursement during the study period were reported. Means were calculated only for those people with at least one reimbursement during the period considered. The total and mean lengths of stay during the 365 days prior to death per patient in the same type of hospital were determined. Percentages were compared by chi‐squared test, ANOVA or Student's *t* test, and medians were compared by Wilcoxon's test or Kruskal‐Wallis test. A Sankey diagram was used to illustrate patient flow according to the presence and the types of hospitalization during the year or during the last 28 days before death.

SAS software (version 7.11, SAS Institute Inc) was used for statistical analysis and R software (3.4.3.) was used for Sankey diagram.

## 3 RESULTS

### 3.1 Patient characteristics

A total of 15 361 individuals who died in 2015 and who were managed for active CRC during the previous year (2014‐2015) were included (Table 1). These individuals had a mean age of 75 years (SD: 12.5 years), 43% were 80 years or older, and 44% were women. Almost 66% of these people died in short‐stay hospitals, 9% died in HaH, 4% died in Rehab, 6% died in SNH, and 15% died at home. On average, these individuals were younger when they died in SSH or Rehab (73 years in both cases) and older when they died at home (76 years), in HaH (78 years) or in SNH (86 years).

**Table 1:** Characteristics, comorbidities, and treatments of patients managed for colorectal cancer during the year before their death

N (row %)	Total	Place of death					*P*	Hospital palliative cares		*P*
		Home	SNH	HaH	Rehab	SSH		Not	Yes	
	15 361	2327	877	1363	692	10 102		6079	9282	
	100.0	15.1	5.7	8.9	4.5	65.8		39.6	60.4	
	%	%	%	%	%	%		%	%	
Characteristics										
Women	44.2	42.3	64.3	53.3	48.1	41.3	***	42.2	45.4	***
Mean age (y) ± SD	75.1 ± 12.5	76.3 ± 12.2	86.5 ± 7.0	78.2 ± 11.1	72.7 ± 12.9	73.5 ± 12.5	***	77.1 ± 11.9	73.7 ± 12.8	***
<60	11.5	9.6	0.1	6.7	13.7	13.4	***	8.2	13.6	***
60‐69	20.5	18.4	3.1	14.6	24.1	23.0		18.0	22.1	
70‐79	25.3	25.2	10.5	25.5	26.7	26.5		24.5	25.9	
80‐89	32.0	34.2	50.4	40.0	28.5	29.2		35.4	30.0	
≥90	10.7	12.6	35.9	13.2	6.9	7.9		13.9	8.6	
Comorbidity										
Lung cancer	2.0	2.1	0.8	1.3	1.8	2.2	***	2.0	2.0	
Prostate cancer	5.6	5.7	7.3	6.7	3.8	5.6		5.5	5.7	
Breast cancer	4.1	4.2	2.5	4.5	4.0	4.3		3.1	4.7	
Other cancers	22.6	20.8	14.7	22.8	23.6	23.5		19.4	24.7	***
Cardiovascular/neurovascular disease	49.2	46.4	61.0	49.3	39.6	49.5	***	56.2	44.6	***
Diabetes	21.5	23.3	20.8	21.0	20.8	21.2		23.3	20.3	***
Mental illness	6.6	7.8	16.6	7.4	6.6	5.4	***	7.4	6.2	*
Neurological or degenerative	12.7	13.5	45.5	13.4	9.0	9.9	***	15.4	11.0	***
Chronic respiratory disease	21.3	19.2	17.9	18.4	18.4	22.6	***	23.8	19.6	***
Chronic inflammatory disease	3.6	3.1	3.4	3.1	3.3	3.8		3.7	3.5	
Rare diseases	0.5	0.5	0.3	0.6	0.6	0.5		0.5	0.5	
HIV or AIDS	0.3	0.2	0.3	0.1	0.1	0.3		0.2	0.3	
Chronic dialysis	0.7	0.6	0.3	0.5	0.3	0.8		1.1	0.5	***
Liver or pancreas	13.3	7.6	4.2	9.3	8.5	16.2	***	12.2	14.0	***
Treatments										
Psychotropic drugs	40.5	44.1	52.5	41.8	44.5	38.2	***	44.5	45.1	***
Hypnotics	18.3	19.7	22.1	17.7	18.8	17.7	*	18.0	18.5	***
Anxiolytics	27.9	30.3	37.4	29.5	31.1	26.2	***	26.9	28.6	***
Antidepressants	19.2	22.0	38.1	19.3	19.2	16.9	***	20.0	18.7	**
Antihypertensives	62.3	64.4	66.4	63.9	59.2	61.5	***	66.2	59.8	***
Lipid‐lowering drugs	29.5	30.4	23.3	31.1	29.3	29.7	***	32.7	27.5	***
At least one stay										
SSH	98.3	93.3	89.7	98.1	99.4	100.0	***	96.0	99.8	***
Total mean length of stay (SD)^a^	52.3 ± 44.1	39.0 ± 36.0	34.7 ± 33.5	53.0 ± 37.0	49.8 ± 37.9	56.5 ± 46.7	***	41.0 ± 41.3	59.3 ± 44.4	***
HaH	11.4	4.6	1.9	100.0	6.4	8.3	***	2.6	17.1	***
Rehab	27.0	19.3	34.5	15.3	100.0	19.1	***	21.1	30.9	***
All types^b^	98.5	93.9	90.3	100.0	100.0	100.0	***	96.2	100.0	***
Total mean length of stay (SD)^a^	71.1 ± 68.1	48.8 ± 54.5	56.1 ± 64.9	109.1 ± 76.0	105.4 ± 87.2	70.1 ± 65.5	***	51.4 ± 57.4	83.5 ± 68.7	***
SNH	9.7	2.9	100.0	6.6	2.3	4.7	***	14.6	6.6	***
Palliative care^c^	60.4	30.0	31.2	94.4	82.1	64.7	***	0.0	100.0	***
Other than at the end of life	9.6	30.0	31.2	2.5	4.3	4.2	***	0.0	15.9	***

a Among people with at least one stay.

b SSH, Rehab, or HaH stays.

c The presence of palliative care at the time of death at home or in SNH cannot be identified.

*** *P* < .001,

** *P* < .01,

* *P* < .05.

At least one other active cancer or a cancer under surveillance was identified for one‐third of these people, with a lower frequency for those who died in SNH (25%) and a higher frequency for those who died in HaH or SSH (35%). Almost one‐half of people presented cardiovascular comorbidity, 22% had diabetes, 21% had chronic respiratory disease, and 13% had a neurological or degenerative disease. These frequencies differed according to the place of death with, for example, 46% of neurological or degenerative diseases and 61% of cardiovascular diseases for people who died in SNH, who were generally older. The 60% of people who received HPC during the year before or at the time of death were slightly younger (74 years vs 77 years) and had fewer comorbidities, but more commonly presented another active cancer or a cancer under surveillance (37% vs 30%).

### 3.2 Hospitalizations and care pathways

**Figure 1:**
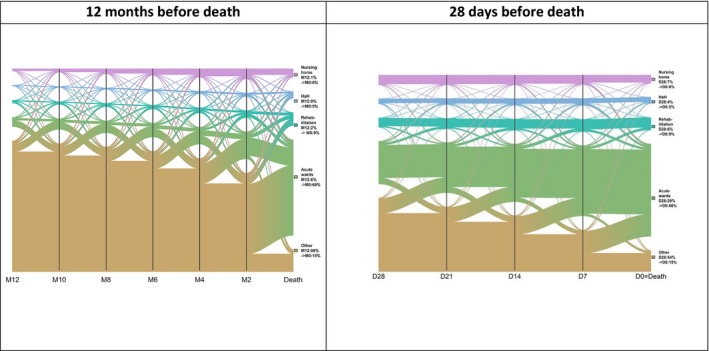
Hospital and skilled nursing home pathways of people managed for colorectal cancer during the year and the 4 weeks before their death in 2015

During the year before death, almost all individuals were hospitalized at least once: 98% in SSH (an average of 52 days during the year), 27% in Rehab, 11% in HaH, and 10% in SNH (Table 1). The mean all‐cause annual cumulative length of stay was 71 days. Among the patients who received HPC, 17% had received this care before prior to the end‐of‐life admission. The mean annual length of hospital stay was 84 days for individuals with HPC vs 51 days for individuals without HPC. Figure 1 illustrates the flows of people between the various institutions during the year before death. These flows were greater during the last 2 months of life, predominantly corresponding to transfers from home to SSH and, to a lesser degree, from SSH to Rehab. By focusing on the last 28 days of life, flows from home to SSH increased before death, especially during the last week: 54% of people were still at home at the beginning of the last month of life and 15% were still at home at the time of death; 29% were in SSH at the beginning of the last month of life compared to 66% at the time of death. These flows were relatively stable for HaH and SNH.

### 3.3 Intensity of medical end‐of‐life care

During the month before death, 83% of individuals were admitted at least once to SSH (68% and 62% of those who died in Rehab or HaH, 46% at home, and 28% in SNH) (Table 2). The mean number of stays during the last month of life was 1.8 and 12% of individuals had two or more stays. The mean length of stay was 17 days. Hospitalization rates and the number of stays decreased with age. Eighty‐eight percent of the 60% of individuals who received HPC vs 75% of those without HPC were admitted to SSH at least once during the last month. Fifteen percent of individuals in Rehab had at least one hospital admission and this frequency increased with age (>60 years: 9%, 80 years or older: 19%) and was higher in the presence of HPC (19% vs 10%). Nine percent of individuals in HaH had at least one hospital admission, especially younger individuals (<60 years: 15%) and in the presence of HPC (14% vs 2%).

**Table 2:** Presence of at least one of the various indicators of intensity of care during the 30 days before death in 2015 for patients managed for colorectal cancer

	Total	Place of death					Hospital palliative care		Age‐group				
		Domicile	SNH	HaH	Rehab	SSH	Not	Yes	<60 y	60‐69 y	70‐79 y	80‐89 y	≥90 y
N	15 361	2 327	877	692	1 363	10 102	6079	9282	1766	3145	3882	4930	1638
%	100.0	15.1	5.7	4.5	8.9	65.8	39.6	60.4	11.5	20.5	25.3	32.1	10.7
SSH stay													
At least one stay	83.2	46.3	27.9	62.0	67.6	100.0	75.2	88.4	92.4	90.6	86.3	78.0	67.0
Number of stays^a^	1.8 ± 1.3	1.8 ± 1.8	1.2 ± 0.9	1.6 ± 1.4	1.5 ± 1.4	1.8 ± 1.3	1.8 ± 1.5	1.8 ± 1.3	2.1 ± 1.5	2 ± 1.6	1.8 ± 1.4	1.6 ± 1	1.4 ± 0.8
Duration (day SD)^a^	17.2 ± 10	11 ± 9.6	13.1 ± 9.6	15 ± 9.8	17.8 ± 9.3	18 ± 9.8	14.4 ± 10.1	18.8 ± 9.6	17.8 ± 10	17.7 ± 10.1	17.5 ± 9.9	16.9 ± 10	15.4 ± 9.6
Rehab stay													
At least one stay	15.1	6.2	8.7	2.2	100.0	7.2	9.7	18.7	9.0	10.8	14.4	19.4	19.0
HaH stay													
At least one stay	9.4	2.1	0.8	100.0	3.5	6.4^b^	1.7	14.4	14.9	11.6	9.9	7.1	4.6^b^
Emergency department admission													
At least one admission	39.1	15.8	15.7	25.6	29.9	48.7	38.1	39.8	36.2	39.1	38.2	40.5	40.4
Followed by hospitalization	38.4	14.5	15.3	24.9	29.6	48.0	37.3	39.1	35.4	38.2	37.2	39.9	40.0
Number of visits^a^	1.2 ± 0.5	1.1 ± 0.4	1.1 ± 0.3	1.1 ± 0.4	1.1 ± 0.3	1.2 ± 0.5^b^	1.2 ± 0.5	1.2 ± 0.5^b^	1.2 ± 0.3	1.2 ± 0.4	1.2 ± 0.3	1.1 ± 0.3	1.1 ± 0.3^b^
Stay for organ failure													
At least one stay	16.5	3.8	1.9	5.3	7.6	22.6	26.7	9.8	16.8	18.0	19.5	15.1	10.6
Chemotherapy													
During last 2 wk													
Hospital session	7.8	7.2	0.8	6.2	6.1	8.9	9.7	6.6	13.3	12.8	9.7	3.6	0.5
Retail pharmacy	1.5	2.7	1.1	0.3	0.1	1.6	2.3	1.0	2.2	2.1	1.4	1.1	1.1
During last 30 d													
Hospital session	14.7	12.5	1.0	10.7	9.8	17.3	15.4	14.3	26.8	22.8	18.2	7.0	1.2
Retail pharmacy	4.7	5.2	2.5	2.2	0.6	5.5	5.8	3.9	6.9	7.0	4.5	3.4	2.0
Radiotherapy (session)													
During last 2 wk	1.6	0.7	0.0	1.4	1.6	1.9	1.3	1.7	3.0	2.6	1.7	0.7	0.4
During last 30 d	2.5	1.6	0.1	2.9	3.1	2.8	1.7	3.0	4.7	4.1	2.4	1.4	0.4

a Among those people with at least one use.

b Not significant.

Thirty‐nine percent of individuals presented at least one emergency department admission during the last month of life, with a higher rate for individuals who died in SSH (49%). A high proportion of people were hospitalized in the context of these emergency department admissions. Seventeen percent of patients (23% in SSH) were admitted to intensive care. At least one chemotherapy session was administered during the last month of life in 15% of patients (17% in SSH and 13% at home), and 8% during the last 2 weeks. These frequencies decreased markedly with age. A reimbursement for retail pharmacy‐dispensed oral chemotherapy was identified for 5% of individuals during the last month of life and for 2% of individuals during the last 2 weeks. Globally, radiotherapy sessions were uncommon (3% during the last 30 days and 2% during the last 2 weeks). Patients with HPC, compared to those without HPC, were more often hospitalised in SSH (88% vs 75%), Rehab (19% vs 10%), and HaH (14% vs 2%). They had a similar frequency of emergency department admissions (39%), slightly fewer chemotherapy sessions during the last 2 weeks (7% vs 10%) and fewer intensive care stays (10% vs 27%).

### 3.4 Causes of death

Cancer was reported as the main cause of death for 84% of the 14 370 people included in this analysis (93% of the study population) (Table 3). This proportion varied according to the place of death: close to 90% in Rehab or HaH, 85% in SSH, 77% at home, and 74% in SNH, 92% for people receiving HPC, and 72% for those not receiving HPC. This proportion decreased with age (<60 years: 94%, 90 years: 74%). The second leading cause of death was cardiovascular disease, accounting for 5% of deaths. A cardiovascular cause of death was more frequent for people who died at home (8%), in SNH (7%) and in the very elderly (10% for people 90 years and older). The third group of causes of death was ill‐defined conditions (3%), including 8% of cases at home and 7% in SNH. More detailed analysis of the group of cancers showed that colon cancer was the main cause of death in 46% of cases and rectal cancer was the main cause of death in 18% of cases, that is, CRC was the cause of death in 64% of cases. Cancers of the Larynx, trachea, bronchus, and lung group accounted for 3% of all deaths and the Other category accounted for 10%. According to the place of death, CRC was the cause of death in 55% of individuals who died at home, 71% of those who died in Rehab, and 57% of those who received HPC. Other cancers accounted for 20% of all deaths (home: 22%, HPC; 21%, and about 22% for people <80 years). When exclusively considering patients with CRC as the cause of death, they predominantly died in hospital: SSH (68% vs 66%), especially in the context of HPC (68% vs 61%).

**Table 3:** Causes of death among people managed for colorectal cancer who died in 2015

All causes	Total	Place of death						Hospital Palliative Care			Age					
		Home	SNH	HaH	Rehab	SSH	*P*	No	Yes	*P*	<60	60‐69	70‐79	80‐89	≥90	*P*
N (%)	14,370 (100)	2,091 (14.6)	806 (5.6)	625 (4.3)	1,297 (9.0)	9,551 (66.5)		5,629 (39.2)	8,741 (60.8)		1,588 (11.1)	2,976 (20.7)	3,677 (25.6)	4,622 (32.1)	1,507 (10.5)	
Cancers	84.1	77.4	74.3	89.6	89.3	85.4	***	71.8	92.1	***	93.5	89.1	85.3	80.2	73.6	***
Cardiovascular	4.9	7.8	6.7	1.9	2.9	4.6		9.2	2.1		1.1	2.4	4.4	6.6	9.5	
Ill‐defined conditions	3.3	7.0	8.4	5.4	2.5	2.0		4.8	2.3		2.5	2.9	2.7	3.5	5.4	
Gastrointestinal system	2.1	0.8	0.9	0.2	1.1	2.7		4.1	0.8		0.8	2.0	2.1	2.6	2.2	
Respiratory system	1.4	1.3	1.6	0.8	1.0	1.5		2.6	0.7		0.6	0.8	1.1	2.0	2.6	
External causes	1.3	2.7	1.2	0.6	0.7	1.1		2.5	0.5		0.4	1.1	1.5	1.3	1.9	
Neurodegenerative	0.7	0.7	3.2	0.5	0.4	0.5		1.1	0.4		0.4	0.5	0.5	1.0	1.1	
Endocrine	0.7	1.3	0.7	0.5	0.8	0.5		1.3	0.3		0.3	0.5	0.7	0.9	0.9	
Infectious	0.6	0.2	0.5	0.2	0.2	0.7		1.0	0.3		0.2	0.4	0.7	0.6	0.7	
Genitourinary	0.4	0.2	0.2	0.2	0.1	0.4		0.6	0.2		0.0	0.2	0.3	0.5	0.8	
Other	0.5	0.6	2.3	0.1	1.0	0.6		1.0	0.3		0.2	0.1	0.7	0.8	1.3	
Cancer details																
Colon	45.9	41.4	39.8	50.9	49.4	46.6	***	38.4	50.7	***	48.1	46.4	46.1	45.5	43.4	***
Rectum, anus	17.7	13.9	17.4	15.7	21.2	18.3		13.6	20.4		23.5	20.5	16.5	16.2	14.1	
Larynx, trachea, bronchus, lung	3.1	2.8	1.5	3.4	3.5	3.2		2.9	3.3		3.9	4.5	3.8	2.1	1.0	
Pancreas	1.3	1.0	0.0	1.8	1.3	1.4		0.8	1.6		1.7	1.5	1.8	0.9	0.3	
Liver	1.3	1.6	1.0	1.4	0.8	1.2		1.3	1.2		1.4	1.5	1.7	1.0	0.4	
Breast	0.9	1.2	0.9	0.6	1.1	0.9		0.7	1.1		1.0	0.7	1.3	0.8	0.9	
Stomach	0.8	0.9	0.1	0.8	0.4	0.9		0.7	0.9		1.2	1.1	1.1	0.6	0.2	
Lymphatic tissue	0.8	0.5	0.5	0.6	0.7	0.8		0.9	0.6		0.5	0.4	0.9	0.8	0.7	
Bladder	0.6	0.7	0.2	0.4	0.4	0.6		0.6	0.6		0.4	0.6	0.5	0.7	0.5	
Prostate	0.6	0.5	1.0	0.6	0.3	0.5		0.6	0.6		0.2	0.3	0.6	0.7	0.9	
Ovary	0.5	0.4	0.2	1.2	0.4	0.6		0.4	0.6		0.7	1.0	0.6	0.3	0.1	
Esophagus	0.4	0.2	0.0	0.0	0.5	0.5		0.4	0.5		0.5	0.8	0.7	0.2	0.0	
Body of uterus	0.3	0.3	0.6	1.0	0.5	0.3		0.2	0.5		0.2	0.4	0.3	0.5	0.4	
Kidney	0.3	0.5	0.5	0.4	0.4	0.3		0.2	0.4		0.3	0.3	0.3	0.3	0.1	
Other, not specified	9.5	11.5	10.6	10.8	8.4	9.4		10.0	9.1		10.1	9.1	9.0	9.6	10.4	
Cause of death: colorectal cancer (n = 9,139, %)	(100.0)	(12.7)	(5.0)	(4.6)	(10.0)	(67.8)	***	(32.0)	(68.0)	***	(12.4)	(21.8)	(25.2)	(31.2)	(9.5)	***

*** *P* < .001.

### 3.5 Cost of the last year of life

**Figure 2:**
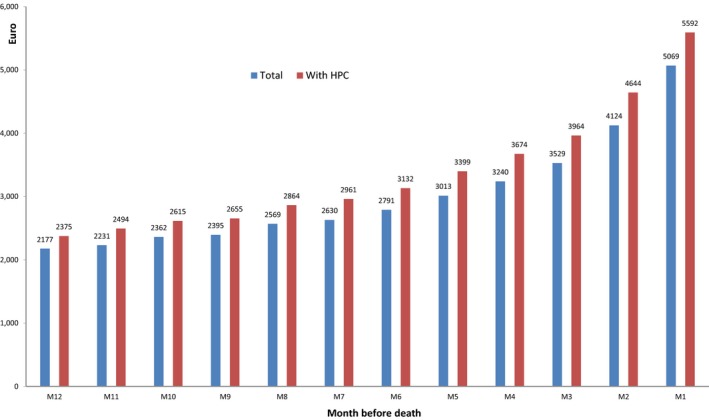
Time‐course of mean monthly national health insurance expenditure during the last year of life among people managed for colorectal cancer who died in 2015 and details concerning the presence of hospital palliative care

The overall national health insurance expenditure for the management of all people with CRC included in this study during their last year of life was €666 million (€449M with HPC, €217M without HPC, including €477M for hospital expenditure, €173M for private practice expenditures, and €16M for other health‐care benefits). The mean annual expenditure during the last year of life was €43,398 (with HPC: €48,804, without HPC: €35,754). The mean monthly expenditure accelerated during the last months of life (Figure 2). The mean monthly expenditure was always higher in the presence of HPC, even during the first few months, with an increasing expenditure toward the end of life.

## 4 DISCUSSION

Few studies have specifically reported the management of patients with CRC 1 year before death on such a large sample size (more than 15 000). Their care pathways involve several types of hospitals with high flows during the last month of life, going from home to SSH, which was the main place of death (66%), and a mean annual length of all‐cause hospital stay of 71 days. An indicator of palliative care was identified in about 61% of all patients managed for CRC and 68% for those with CRC as the cause of death. Emergency department admissions and chemotherapy sessions were frequent during the last month of life, especially among the youngest patients with fewer comorbid conditions. The main cause of death was a cancer for 84% of patients and CRC for 64% of patients, with variations according to age, place of death, and HPC management. The mean annual national health insurance expenditure per person at the end of life was €43 000.

A similar study, but based on all cancer patients in France (mean age: 73 years) who died in 2013, revealed 67% of deaths in SSH, 8% in Rehab, 4% in HaH, 5% in SNH, and 15% at home or other places.^22^ These proportions were similar to those observed in the patients of this study (mean age: 75 years) managed for CRC, with 66%, 9%, 4%, 6%, and 15%, respectively. The end‐of‐life management of cancer patients in France, including patients with CRC, is hospital‐centered, as the 67% of patients with all types of cancer who died in SSH is higher than that reported in seven countries in 2010, ranging from 29% in The Netherlands to 54% in Canada.^24^ Another study conducted in six European countries concerning the proportion of cancer deaths at home in 2003 revealed proportions ranging from 12% in Norway to 45% in The Netherlands,^25^ that is predominantly higher than the proportions observed in France with only 15% of deaths at home or 19% when taking HaH into account. More specifically, a Canadian study on patients with CRC as cause of death (2006‐2010), reported 50% of deaths in SSH, a lower proportion than that observed in this study, with variations according to the zone of residence.^15^ The proportion of each place of death and the mode of management can vary according to the definition of the cases included, either people who died while being managed for cancer or people for whom cancer was the main cause of death, the study period, but also according to age, the type of cancer, its stage and its history, and comorbidities, including associated cancers, as well as the development and organization of palliative care.^10^, ^11^, ^12^, ^13^, ^14^, ^15^, ^16^, ^17^ Observed frequencies vary according to organization of end‐of‐life care or palliative care in each country, either mainly in short‐stay hospitals, as in France, or by preferring discharge from hospital and management at home or in hospices. In France, nursing home care or hospice care are not developed as in countries with management at home or in a hospice before dearth, as in the United States where an increasing number of deaths in hospices has been reported (42% of patients 65 years and older with cancer, chronic obstructive pulmonary disease, or dementia in 2009) or Taiwan (47% in 2010‐2).^26^, ^27^ In France, 5% of cancer patients died in SNH and 9% died in Rehab, two structures that can be considered to resemble hospices.^22^ Nevertheless, a Canadian study, in front of a less hospital‐centered organization and aggressive treatments use recommended the need for a major reconceptualization of death, dying, and end of life care to ensure sufficient capacity of palliative home care.^28^

Few published studies have described the indicators of care for CRC. In the all‐cancer study comparing seven countries in 2010, the proportion of people with at least one SSH admission during the last month of life ranged from 44% in The Netherlands to 64% in Norway, lower than the 84% of SSH admissions for CRC patients observed in our study, which also revealed a high rate of multiple admissions, but also a high proportion of deaths in SSH (43%).^24^ The rates of at least one ICU admission, for all cancers, ranged from 9% in Germany to 18% in Belgium, and was 15% for CRC in this study.^28^, ^29^ Emergency department admissions were reported for 27% of cases in Germany to 58% of cases in England and 42% of cases of CRC in France.^24^ The presence of chemotherapy ranged between 24% in Norway and 41% in Belgium, higher than in France with 17% for CRC during the last month of life. A French study specifically devoted to chemotherapy in a population of patients with metastatic cancer who died between 2010 and 2013 reported a frequency of 19% during the last month of life (11% during the last 2 weeks).^30^ Factors associated with the use of chemotherapy were young age, few comorbid conditions, a chemosensitive cancer with significant life expectancy, absence of HPC in the hospital, and the private sector. More specifically, in Canadian patients with CRC, the frequencies of these various indicators were lower than in France: 4% received chemotherapy during the last 2 weeks of life, 12% attended an emergency department, 10% had multiple hospitalizations, and 2% were admitted to ICU.^15^ Another North American study reported a marked increase in the use of chemotherapy in patients 65 years and older with metastatic CRC between 2000 and 2009, with no survival benefit in patients 75 years and older.^16^ In Italy, in a study conducted between 2007 and 2014, almost 10% of patients had initiated a new chemotherapy protocol during the last month before death and 7% of patients with metastatic CRC had received chemotherapy during the last 2 weeks before their death, figures similar to those observed in our study, in which 10% of patients received IV or oral chemotherapy.^10^ Factors associated with these late chemotherapy prescriptions, which can be considered to be inappropriate, were, as in our study, younger age and the absence of previous prescription of a treatment considered to be active.^10^ The use of oral chemotherapy during the last month of life, observed for almost 5% of patients, especially those who died at home or in SSH, could increase in the future with the growing use of targeted therapies such as regorafenib or trifluridine‐tipiracil as third‐ or fourth‐line treatment for colon cancer.^31^ All of these indicators of aggressive end‐of‐life management were higher in people under the age of 65 years compared to older people, as also reported in the United States.^32^ In contrast, chemotherapy was used less frequently in patients receiving HPC, which is consistent with palliative care, which is initiated relatively late in France, often coinciding with discontinuation or limitation of anticancer drug treatments.

The use of palliative care, especially at an early stage, had a positive impact on the patient's quality of life and was associated with decreased intensity of medical health‐care utilization and decreased use of aggressive treatments during the last month of life.^11^, ^17^ In this study, the presence of HPC during the year preceding death or at the time of death was identified for 60% of patients. As in other studies, patients managed by palliative care were younger and presented fewer comorbidities.^5^, ^11^ In the present study, patients managed by HPC more often presented a cancer as cause of death (97%). However, CRC was the cause of death for only 70% of patients managed by HPC, also reflecting the large proportion of other associated cancers. Nevertheless, people managed by HPC were more often hospitalized in the various types of hospital units, with a higher mean annual length of stay, a higher rate of hospital admissions during the last month of life, a similar rate of emergency department admissions and less frequent intensive care unit admissions. These findings can be explained by the hospital‐centered end‐of‐life management in France. However, 30% of patients treated for CRC who died at home or in SNH had received HPC. Analysis of those cases for which CRC was the cause of death revealed a higher rate of palliative care use in these patients (68% vs 61%), by eliminating other noncancer causes of death. However, analysis of the impact of HPC on the intensity of care during the last year or the last month of life is limited, as the great majority of HPC was delivered during the end‐of‐life stay for specific patient groups.

The estimated number of CRC‐related deaths, based on French cancer registries, was 9200 in metropolitan France in 2018.^18^ In the present study, CRC was the main cause of 7700 deaths for 77% of the population, corresponding, by crude extrapolation, to a larger sample size of 10 000 deaths, but for all of France in 2015, bearing in mind that the characteristics of populations not included on the basis of their national health insurance scheme are probably different. Another approach to estimating the representativity of the population included in this study is to link causes of death and active cancers or cancers under surveillance by means of specific algorithms. Thus, the active CRC inclusion algorithm identified 84% of all deaths due to CRC in the study population. Among the remaining 16% of people with CRC as the main cause of death, 6% were identified by algorithms as presenting another active cancer, 7% as presenting a cancer under surveillance, and 3% as not presenting any cancer. The presence of other active cancers can be explained by the frequent association of several different cancers, as one‐third of people with active CRC in our study were actively treated or followed for another cancer.

The distribution of the causes of death among people with active CRC identified in our study was as follows: CRC: 64%, other cancers: 20%, and noncancer causes: 16%. An American study linking patients from a cancer registry to their causes of death (1973‐2012)^33^ revealed a decreased proportion of patients with CRC as cause of death (70% in 1970 to about 45% in 2012), a stable percentage of about 10% for other cancers, but a higher proportion of noncancer causes of death, stable at around 45%. The differences between these two studies are undoubtedly related, among other factors, to the different epidemiological contexts between these two countries.

Few studies have reported the expenditure related to the last year of life for such a large population of individuals managed for CRC. A German study on the direct cost of palliative care, based on reimbursement data after treatment, reported a mean cost of slightly more than €30 000, lower than that observed in our study (€43 398).^34^ A Finnish study reported a mean half‐yearly cost of about €20 000 for metastatic CRC and the cost of end‐of‐life care was similar to the annual cost of the last year of life in this study.^35^ Rapidly instituted palliative care is associated with decreased costs related to a shorter hospital length of stay.^36^ In this study, patients managed by HPC presented a higher mean cost. It is difficult to assess the possible impact of HPC in our study, as HPC was delivered to only a small proportion of people before the end‐of‐life stay and the patients managed by HPC, with more advanced disease, probably presented more intense health‐care consumption, already observed one year before death.

The main strengths of this study are the use of the SNDS population database comprising the health‐care use of almost 77% of the French population. Diseases can be identified by multisource algorithms such as those used in this study. However, the algorithms depend on the use, offer, and access to care. Nevertheless, there is a good concordance between algorithms and causes of death for CRC. The absence of information on the stage and documented presence of metastases, as well as the presence of other cancers, prevents us from precisely examining whether health‐care utilization was elective or delivered in the emergency setting, or whether certain health care was associated with the specific management of CRC. The health‐care use indicators described cannot be used to reliably determine the appropriate or inappropriate nature of this care. Certain clinical factors and certain personal preferences may or may not justify the same type of care or an intercurrent event resulting in death and emergency room diagnosis are not available. This study exclusively concerned patients with active cancer 1 year before death, but this cancer may have been diagnosed and treated over a short period before death.

The overall use of palliative care, including HPC, is probably underestimated because this study did not take into account private practice or SNH palliative care, which cannot be identified by our database. The data analyzed in this study were derived from administrative databases with their classical limitations concerning their primary objective, that is data collection and coding.

This study suggests a relatively high level of hospital with HPC use in France for people with CRC during the year before death. In the context of a reflection on the end of life, these results must be refined in order to elucidate various specific aspects (diseases, disparity of use, etc), but also with the help of health‐care professionals and patients, in order to guide end‐of‐life health policies and evaluate heath policies and improve monitoring and assessment of HPC use.

## CONFLICT OF INTEREST

The authors declare that they have no competing interest.
